# Copy number variation in the bovine genome

**DOI:** 10.1186/1471-2164-11-284

**Published:** 2010-05-06

**Authors:** João Fadista, Bo Thomsen, Lars-Erik Holm, Christian Bendixen

**Affiliations:** 1 Group of Molecular Genetics and Systems Biology, Department of Genetics and Biotechnology, Faculty of Agricultural Sciences, Aarhus University, Blichers Allé 20, DK-8830 Tjele, Denmark

## Abstract

**Background:**

Copy number variations (CNVs), which represent a significant source of genetic diversity in mammals, have been shown to be associated with phenotypes of clinical relevance and to be causative of disease. Notwithstanding, little is known about the extent to which CNV contributes to genetic variation in cattle.

**Results:**

We designed and used a set of NimbleGen CGH arrays that tile across the assayable portion of the cattle genome with approximately 6.3 million probes, at a median probe spacing of 301 bp. This study reports the highest resolution map of copy number variation in the cattle genome, with 304 CNV regions (CNVRs) being identified among the genomes of 20 bovine samples from 4 dairy and beef breeds. The CNVRs identified covered 0.68% (22 Mb) of the genome, and ranged in size from 1.7 to 2,031 kb (median size 16.7 kb). About 20% of the CNVs co-localized with segmental duplications, while 30% encompass genes, of which the majority is involved in environmental response. About 10% of the human orthologous of these genes are associated with human disease susceptibility and, hence, may have important phenotypic consequences.

**Conclusions:**

Together, this analysis provides a useful resource for assessment of the impact of CNVs regarding variation in bovine health and production traits.

## Background

Cattle, part of the Cetartiodactyl order of eutherian mammals [1], is an important source of human nutrition worldwide as well as the most studied ruminant model of metabolism, reproduction, and disease [2]. Following the milestone publication of the cattle genome assembly along with annotation of functional elements and variation [2,3], we are now enabled to search for genomic regions that impact the genetic variation of important phenotypic traits.

Genomic structural variation, including insertions, duplications, deletions, inversions and translocations of DNA, has long been known to be present in animal genomes [4,5] but had predominantly been assumed to be to rare events and often associated with disease. This notion changed in 2004 when two groups of researchers published the first genome-wide maps of copy number variation in seemingly healthy individuals [6,7]. Copy number variant (CNV) is described as a segment of DNA > = 1 kb that is copy number variable when compared with a reference genome [8]. Before these landmark studies, it was thought that SNPs were the major source of genetic variation between individuals [9] but genomic structural genetic variation is now known to cover more base pairs [10-17], and to have a higher per-locus mutation rate than SNPs do [18].

There are indications that CNVs appear throughout the genome not only in humans, but also in other primates [19-21], rodents [22-30], flies [31,32], dogs [33], chickens [34] and cattle [35]. Nevertheless, other than humans and mice [29,36-40], little is known about how CNVs contribute to normal phenotypic variation and disease susceptibility. Up until now, relatively few studies have confirmed the presence of CNVs in cattle [35,41,42], of which only one study focused on genome-wide detection of CNVs [35], but at low resolution using version 3 of bovine genome assembly [2].

Here we report the use of high-resolution oligonucleotide array comparative genomic hybridization (array CGH) to identify 304 CNV regions in 20 animals (14 Holsteins, 2 Red Danish, 3 Simmental and 1 Hereford). With an average probe spacing of 420 bp relative to the latest bovine genome assembly (BT4, 2007) [2], this analysis provides the highest-resolution map of copy number variation in the cattle genome to date.

## Results

### Experiment design

The goal of our study was to characterize levels and patterns of copy number variation among bovine animals. Therefore, to assess the bovine CNV landscape, the genomic DNA of 20 bovine samples from two dairy (14 Holsteins, 2 Red Danish) and two beef breeds (3 Simmental, 1 Hereford) were analyzed. Assessment of copy number variation between samples was done using a set of Nimblegen HD2 CGH arrays that tile across the genome with approximately 6.3 million unique oligo probes with a mean probe spacing of 420 bp, using the latest genome assembly (BT4) [2].

We opted for a dye-swap loop array design, rather than a common reference design, so that each sample was hybridized to two different samples in two different dye orientations. Dye swap is used to compensate for dye bias, while the loop design (known to be more efficient than the reference design [43,44]) is applied to help assign the CNV gain and loss status more accurately for each sample based on the number of samples with the CNV.

### Array CGH evaluation

For evaluation of our array CGH platform, four sex-mismatched arrays and one self-self hybridization (all in dye swaps) were used to assess the false positive rate (see Methods). Probes were interpreted as revealing a copy-number difference if the standard error of the log-intensity ratio was beyond an intensity-ratio threshold. The adequacy of this threshold in detecting copy-number differences was confirmed by conducting sex-mismatched hybridizations, comparing the number of X-linked probes beyond the threshold. From the 88.52 Mb length of chromosome X, 3.21 Mb were greater than the threshold, yielding an estimate of 3.62% for the rate of false positives (FP). The false positive rate (FPR) is conservatively overestimated due to: (1) the assumption that there are no CNVs in the chromosome X of sex-mismatched arrays; (2) calling for FP was done at individual arrays rather than if they were detected in both dye swaps; (3) and because the self-self hybridization array yielded much lower FPR at individual array calling (0.0085%), and zero FPR when calling CNVs detected in both dye swap.

### Pattern and frequency of CNV regions

**Figure 1:**
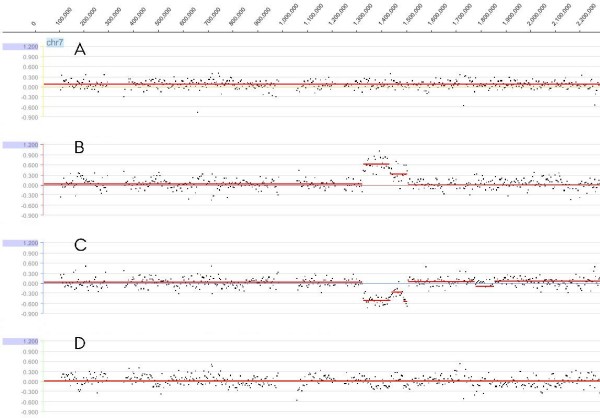
**Example of an identification procedure for CNV gain or loss status**. Y axis represents log2ratios and X axis represents genomic positions along chromosome 7. (A) animals *2 vs. 19*; (B) *19 vs. 6 *(gain in 19 or loss in 6); (C) *6 vs. 20 *(loss in 6 or gain in 20) and (D) *20 vs. 17*. The only plots that show a CNV are B and C, and since the only animal common to those hybridizations is animal 6, we classify this CNV as a deletion in animal 6.

Since copy-number changes are relative for array CGH data, unambiguous ascertainment of the ancestral state of a CNV and (subsequent) identification of duplications and deletions is challenging. We have therefore chosen a design where a dye swap is coupled with a loop design, with each animal sample hybridized with two other animal samples, enabling us to distinguish between a deletion and duplication as well as the animal origin of the CNV (Figure 1). Since identical CNVs, when called in different animals, might be assigned different boundaries due to technical and/or biological sources of variability, overlapping CNVs were handled as a whole and named copy number variable regions (CNVRs) [10,45].

**Figure 2:**
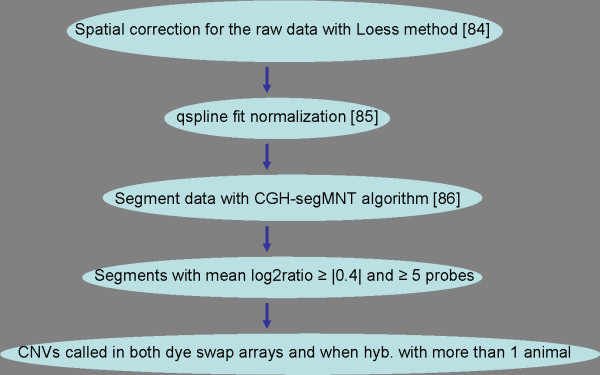
**CNV calling pipeline (details in Methods section)**. Data analysis procedure to discover copy number variations in array CGH data.

After applying a stringent CNV calling pipeline with a theoretical 1.5 kb resolution for CNV detection (Figure 2 and Methods), 304 putative CNVRs were identified, averaging 47 CNVs per animal (Additional file 1, Table S1), with 70% (212) of the CNVRs observed in more than one animal. Although CNVRs detected in more than one animal of different families and/or breeds could be defined as frequent, the relationship between some of the animals precludes such classification (Additional file 2, Figure S1). The relatively poor breakpoint estimation also prevents information regarding whether these CNVRs are identical-by-descent (arisen before the divergence of these cattle breeds), or separate events occurred independently in different breeds (in putative structurally fragile genomic regions).

**Figure 3:**
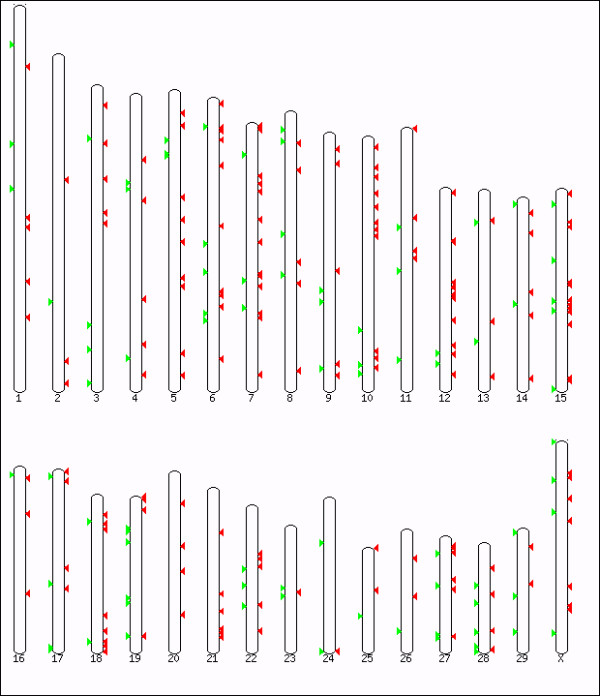
**Bovine karyotype, with CNVR distribution**. Green arrows correspond to gains and red arrows correspond to losses.

CNVRs were detected on all chromosomes, but were distributed throughout the genome in a non-random manner (Figure 3) with little correlation between CNVRs occurrence and chromosome length (Additional file 3, Figure S2). This is coherent with previous studies on heterogeneous distribution of CNVs in primates [10,21], but the low number of samples used in this study prevents us from drawing any conclusions regarding putative genomic CNV hotspots.

**Figure 4:**
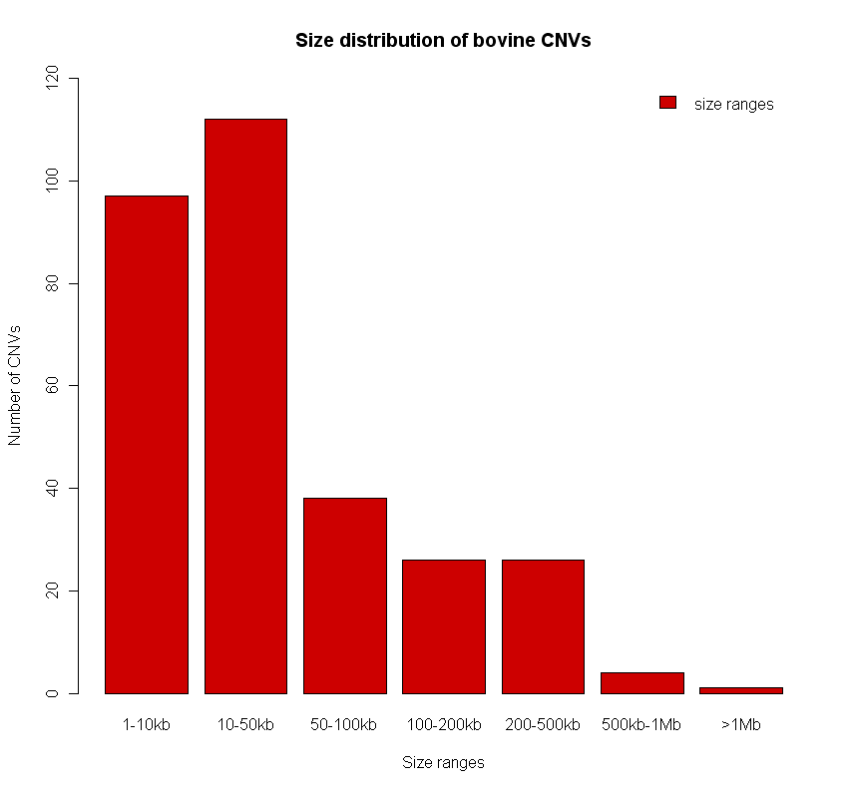
**Size range distribution of the CNVRs detected**.

Out of the total number of CNVRs detected, 49.7% (151) partially overlap gaps in the assembly (BT4), which indicates that the CNVRs have a high probability of being linked with gaps within the reference cattle genome assembly (permutation test, p-value < 0.001). This stresses the need for unraveling of these genomic regions of high structural complexity. The CNVRs detected vary in size from 1.7 kb to 2 Mb with a median size of 16.7 kb, and encompass approximately 23 Mb or 0.68% of the bovine genome (Table 1 and Figure 4). The biggest region showing copy loss is 2.03 Mb on chromosome 13 in animal 16, while the biggest region showing copy number gain was detected in animals 6 and 7 showing a 417 kb amplification without overlapping any gene nor SD (Additional file 1, Table S1).

**Table 1:** Characteristics of the CNV regions, with sizes in base pairs (bp).

Type	CNVRs	Mean size	Median size	Size range	CNVR Content	Sequence covered	% CNVR
Loss	202	77,420	16,543	1,716 - 2,031,343	15,638,770		0.482
Gain	102	62,147	19,580	1,737 - 416,858	6,339,041	3,247,516,410	0.195
All	304	72,295	16,678	1,716 - 2,031,343	21,977,811		0.677

When comparing the size distribution between the 202 losses and the 102 gains, no significant difference was found (Wilcoxon rank sum test, p-value > 0.05), although we detected significantly more losses than gains (exact binomial test, p-value = 1.01e-08). This bias in detecting more deletions could be due to both biological and technical reasons. One of the main mechanisms responsible for the CNV formation, non-allelic homologous recombination (NAHR), has been shown to generate more deletions than duplications [46]. As also noted by others [10,31,47], a technical bias favoring the detection of deletions may be responsible since our CNV detection pipeline have more power to detect a loss (log2(1/2) = -1) than a gain (log2(3/2) = 0.58). With 69% of the CNVRs described within 50 kb in size (figure 4), it should be noted that a significant proportion of the CNVs are near our effective resolution of 1.5 kb. This indicates that the experimental detection of 304 CNVRs may greatly underestimate the actual number of CNVs in the cattle genome, and that a substantial proportion of CNVs could be smaller than 1.5 kb in size. CNVs >2 Mb in size were not detected, which may be a consequence of the number and size of sequence gaps in the current outline of the cattle genome sequence assembly (75 654 gaps spanning 5.8% of the assembly).

When assessing hybridization signals in the unassembled chromosome (ChrUn), it was verified that only the male vs. female hybridizations were detecting CNVs in some regions. Although the number of females in this study is small (n = 2), the findings suggest that these regions may be from the bovine chr Y (Additional file 11, Table S9). It is known that the *Bos taurus *genome assembly was not only composed by a female animal, but also had a BAC library sequenced from a male animal, from which the corresponding Y chromosome scaffolds were unlabeled and placed in the ChrUn [48]. Consequently, this study highlights regions for future genome assembly improvements.

In accordance with analyses conducted in humans [10,11], we detected that the GC content of the CNVRs (43.6%) are slightly larger than of the whole genome (41.8%), which supports the notion that CNVs arise more often in GC rich regions.

### SDs are associated with CNVs

Segmental duplications (SDs), defined as regions of length > = 1 kb with at least 90% sequence identity [49], are important elements in the formation of CNVs via non-allelic homologous recombination (NAHR) throughout the mammalian lineage [39,50,51]. To test whether the non-random association between CNVs and SDs was preserved in our high-resolution data, the overlap of CNVs with segmental duplications was determined. Segmental duplications were overlapped by 20% (61) of the CNVRs, which implies that CNVs are enriched near segmental duplication (permutation test, p-value < 0.001). It should be noted that the enrichment is increased when testing only the CNVRs bigger than 20 kb, with segmental duplications overlapping 47% of those CNVRs. This is also consistent with previous CNV studies reporting a stronger association between segmental duplications and long CNVRs [10,45].

### Functional analysis

Nearly 30% (90) of the CNVRs encompassed 348 full-length genes as annotated in Ensembl [52] (Additional file 4, Table S2), but contrary to segmental duplications, the enrichment of CNVRs in genic areas is not significant. This indicates that the gene content of the CNVRs does not significantly differ from the whole bovine genome. The fact that none of the 481 ultraconserved elements [53], nor the 611 new long conserved noncoding sequences in vertebrates [54], were found in the CNV regions (whereas six of them would be expected by chance, permutation p-value < 0.001), further supports the notion that CNVs are significantly depleted in highly conserved functional elements.

In order to determine the likely biological effects of the 348 copy number variant genes, a gene ontology (GO) analysis was performed with the EasyGO tool [55]. Genes that were not completely included within the CNVRs were excluded from the GO analysis, since the breakpoint definition of CNVRs can be equivocal [56,57]. Table 2 shows that genes involved in environmental response are over-represented in the bovine CNVRs, as also seen in other studies of mammalian genomes [10,21,25,33].

**Table 2:** Enriched GO terms associated with the CNV regions (FDR p-value ≤ 0.01).

Cellular_Component		
GO term	GO name	p-value
GO:0031224	intrinsic to membrane	3.08e-10
GO:0016021	integral to membrane	3.08e-10
GO:0016020	membrane	2.73e-10
GO:0044425	membrane part	2.16e-10

**Molecular_Function**		

GO term	GO name	p-value
GO:0060089	molecular transducer activity	1.36e-29
GO:0004867	serine-type endopeptidase inhibitor activity	0.00448
GO:0004984	olfactory receptor activity	1.6e-26
GO:0004872	receptor activity	8.73e-31
GO:0004871	signal transducer activity	1.36e-29
GO:0004930	G-protein coupled receptor activity	1.6e-26
GO:0005044	scavenger receptor activity	1.53e-06
GO:0004888	transmembrane receptor activity	9.94e-32

**Biological Process**		

GO term	GO name	p-value
GO:0007165	signal transduction	7.62e-16
GO:0007166	cell surface receptor linked signal transduction	3.74e-21
GO:0050789	regulation of biological process	3.84e-10
GO:0065007	biological regulation	9.61e-10
GO:0007186	G-protein coupled receptor protein signaling pathway	1.13e-21
GO:0050794	regulation of cellular process	5.01e-10
GO:0006952	defense response	0.0107
GO:0007154	cell communication	1.82e-15

The following step was to test if genes unaffected by CNVs exhibited a different selective constraint than the ones affected. To test this, the dN/dS ratios for orthologous genes between the cow and human species were compared (Table 3 and Additional file 5, Table S3). Knowing that dN is the number of nucleotide differences per non synonymous site and dS the number of nucleotide differences per synonymous site, dN/dS < 1 suggests that amino acid change is selectively constrained (purifying selection), while dN/dS ≥ 1 suggests a relaxation of that same selection (positive selection). It was determined that both deleted and duplicated genes have dN/dS ratios significantly higher than those for non-polymorphic genes. This result, as for the over represented set of 'environmental response' genes, might indicate a relaxation of constrains due to the redundancy expected from the variable number of gene copies [58-61].

**Table 3:** Evolutionary rates for monomorphic and CNV genes.

	Duplication CNV	Deletion CNV	No CNV
**dN/dS**	0.339	0.392	0.165

**Wilcoxon rank-sum test, P value**	1.39E-06	< 2.2e-16	-

dN - nonsynonymous rate; dS - synonymous rate; p-value compares CNV genes with monomorphic genes.

When examining the human orthologs for the cattle genes [52] affected by CNV, we studied 167 human ortholog genes of which 84 overlap with the genomic coordinates of previously reported human structural variation, as seen in the Database of Genomic Variants (DGV) [7]. Since it is unlikely that CNVs in the human-cow common ancestor would have been conserved, the overlapping CNVs most probably reflect the existence of orthologous genomic regions of structural instability that are prone to recurrently generate polymorphisms in both species. However, this may also indicate that the CNVs annotated in DGV, being derived using different technological and analytical platforms, have a large variance in CNV resolution which may overestimate CNV sizes [14]. Consequently, even if the annotated CNVs represent true structural variation it is difficult to estimate the actual boundaries of the CNV and subsequently the overlap of DGV CNVs with the CNVs identified here.

### CNV affecting genes associated with disease

Querying for copy number variant genes that had an orthologous human gene with OMIM morbid ID reference [62], revealed that 19 of these genes have been associated in human disease (susceptibility to sarcoidoisis and Alzheimer's disease, myopathy, encephalopathy, ataxia, etc - Additional file 6, Table S4). Likewise, when probing orthologous human genes involved in genome-wide association studies (GWAS) [63], 12 genes associated with human disease traits were found (Additional file 7, Table S5). We also queried the Animal QTL database [64] that holds publicly available QTL data on livestock species. Retrieving all the bovine QTLs within 2 Mb of our CNVRs resulted in 110 QTLs, which can hold putative valuable information for some important traits of interest (Additional file 8, Table S6). The database of Online Mendelian Inheritance in Animals (OMIA) [65] was also queried, and 21 cow phenotypes within 2 Mb of CNVRs were retrieved (Additional file 9, Table S7).

### Comparison with other mammalian CNV studies

Next, we compared the number of CNVRs detected here with CNVRs from other studies (Table 4). To minimize technical CNV detection biases we: (1) used data only from the same platform (when available), (2) used data from the highest resolution genome-wide survey published on each species queried and (3) required that the study was non biased to any particular genomic region. The main finding of this comparison is that an increased resolution of the array platform increases the number of detected CNVs. This supports our finding that the bulk of CNVs in mammalian genomes are small events, implying that the characterization of the mammalian CNV landscape is far from complete. A comparison with other non-human CNV studies shows that the number of CNVRs/sample does not follow the same trend. This is expected since we have employed a number of related animals as well as the overall genetic variation in cattle is known to be reduced relative to mouse and man. Another contributing factor might be that our stringent CNV calling criteria hampers the detection of putative true CNVs. Concerning the platform used to assess CNVs in humans [17]; with a resolution to find CNVs 3 times bigger than ours, a similar difference when detecting CNVR/sample would be expected. This is not the case because CNV counts are known to be inversely proportional to their size, as seen here and elsewhere (DGV [7]).

**Table 4:** Comparison between this and other mammalian CNV studies using array CGH.

Species	Samples	CNVRs	CNVR/sample	Mean size	Platform	Mean spacing	Resolution	Ref
Cow	20	304	15	72	3*2.1 M	0.4	2	This
Cow	5	25	5	128	385 k	6	28.8	[35]
Dog	9	60	7	310	385 k	4.7	23.5	[33]
Mouse	20	1333	66	64	2.1 M	1	5	[45]
Rat	3	33	11	256	385 k	5	25	[30]
Macaque	10	123	12	102	385 k	6.5	32.5	[21]
Human	40	8599	215	20	20*2.1 M	0.06	0.5	[17]

Mean size, Mean spacing and Resolution are given in kilobases (kb).

### Validation of the CNVRs

To evaluate the accuracy of the copy number assignments, quantitative real time-PCR was used as described previously [47]. Briefly, two control regions, one site on the X chromosome and one site on an autosome, plus ten potential CNV regions were selected. Six quantitative PCRs immediately confirmed the existence of copy number variation in these regions (Additional file 10, Table S8), whereas primer sets in four regions did not work satisfactorily (see Methods). Primer sets were therefore re-designed within these four CNV regions, and using these new primers the PCR reactions were performed successfully. Thus, the existence of copy number variation in all ten regions was confirmed by quantitative PCR.

## Discussion

The study outlined in this paper yields the highest-resolution analysis of bovine CNVs to date. Using a genome-wide tiling oligo array CGH, the largest number of CNV regions yet reported in cattle (304 CNVRs; average of 47 per genome) have been identified. Almost all (98%) of the CNV regions discovered here are novel relative to previous reports [35,42], thereby vastly expanding our insight of genome structural variation in cattle. With an effective resolution of 1.5 kb in detecting CNVs, resulting in a median CNV size of 16.7 kb, our data shows that at least 0.68% of the cattle genome can vary in copy number in seemingly healthy animals. This is most probably an underestimate of the true genomic fraction that is tolerant to copy number due to the low number of animals sampled and their close relatedness.

As detected previously, not only in cattle [35] but also in other species [10,25,27,33], CNVs are strongly associated with segmental duplications (SDs). This SD relation creates a lack of probe coverage in and around duplicated sequences [66], which significantly hampers the applicability of genome-wide association studies using SNP arrays to tag SDs-driven CNVs.

In addition, our data suggests that smaller CNVs (<50 kb) are much more frequent than larger ones, which is in agreement with other high resolution studies [14,17,45]. If this can be extrapolated for the whole cattle genome, the commercially available Illumina 50 k SNP panel (with an average probe spacing of 54 kb [67]) would not be sufficient to detect the bulk of existing CNVs. Consequently, further characterization of cattle CNVs should be done with similar high-density array CGH or using next-generation sequencing technologies. The latter identifies a more complete size and class ranges of structural variation [68-79].

As previously shown, copy number variants can have an impact on phenotypic variation mainly due to gene-dosage effects [80], and are often associated with disease susceptibility [38-40,81]. In this analysis, CNVRs were found to be enriched for genes with functions related to environmental response, such as immune and sensory functions previously noticed in other species [10,25,27,33,35]. The enrichment aspect is an interesting finding, since variation in immunity related genes have been associated with disease. In particular, genes of the major histocompatibility complex (MHC) http://www.ebi.ac.uk/ipd/mhc/, of which some are included in our dataset, are reported to be responsible for differences in predisposition to diseases like mastitis, dermatophilosis and other tick infections [41]. Concerning the genetics of milk production and lactation, we found none of the 197 unique milk protein genes and the over 6000 mammary-related genes within our CNV regions. This is expected since these genes are known to be highly conserved and evolving more slowly than other genes in the bovine genome [82].

Many genes and QTLs associated with human and cow diseases were found to be copy number variable or located nearby CNV regions. The fact that some bovine CNVs occurred in regions orthologous to human CNVs, reflect most likely recurrent CNV formation, rather than ancestral CNVs maintained in both species. These regions could be hotspots of CNV genesis due to their fragile structural architecture that prompts frequent rearrangements.

## Conclusions

In summary, the data presented here extends and establish the fact that a significant part of cattle genome is copy number variable within and between breeds and that our high-resolution array CGH is a valid method to detect bovine CNVs in a genome-wide manner. With a limited amount of sampled animals and breeds, and a stringent CNV calling criteria, the CNV regions reported here are believed to be highly reliable, but the number might greatly underestimate true number of CNVs in cattle populations. Consequently, future studies are required to assess the functional significance of CNVs and their impact on health and productive efficiency in cattle.

## Methods

### Sample preparation

The genomic DNA of 20 bovine samples was obtained from 4 dairy and beef breeds (14 Holsteins, 3 Simmental 2 Red danish and 1 Hereford). The pedigree scheme for the related animals is in Additional file 2, Figure S1. DNA was extracted and purified from blood as described elsewhere [47], in order to pass Nimblegen quality control requirements. We adhered to our national and institutional guidelines for the ethical use and treatment of animals in experiments.

### Array CGH

DNA fragmentation, labeling, hybridization, washing and array imaging were carried out according to the manufacturer's protocol and done as previously described [83]. Briefly, the genomic DNA samples were fragmented by sonication and labeled with fluorescent dyes Cy3 and Cy5. According to the dye swap loop design (Additional file 12, Table S10), samples were co-hybridized with a MAUI hybridization system (BioMicro Systems) to custom-made cattle CGH 2.1 M (HD2) arrays (Roche NimbleGen, Madison, WI). In order to cover the latest bovine genome assembly (bt4) with high density, the custom CGH arrays were planned in 3 designs. Each design covered a specific set of chromosomes with 2.1 million probes, which yielded 420 bp of average probe spacing (301 bp median probe spacing). The probe design fundamentals are described by the array manufacturer and elsewhere [47].

The arrays were scanned using a 5 μm scanner, and Nimblescan software (Roche Nimblegen, Madison, WI) was used to retrieve fluorescent intensity raw data from the scanned images of the oligonucleotide tiling arrays. For each spot on the array, log2-ratios of the Cy3-labeled test sample versus Cy-5 reference sample were computed. Before normalization and segmentation analysis, spatial correction was applied. Spatial correction reduces some artifacts observed in CGH data from 2.1 M arrays, adjusting position-dependent non-uniformity of signals across the array. Specifically, locally weighted polynomial regression (loess) was used to adjust signal intensities based on X, Y feature position [84]. Normalization was then performed using the q-spline method [85], followed by segmentation using the CNV calling algorithm segMNT [86]. This algorithm is shown to outperform both DNACopy [87], which is one of the most widely used CNV calling algorithm in the literature, and StepGram [88], the algorithm used by Agilent for CGH arrays. The segments with mean log2ratio ≥ |0.4| and at least 5 consecutive probes were retained. From these, a CNV was called if it was detected in both dye swap arrays and detected at least in two different dye swap hybridizations (i.e. in two hybridizations with an animal in common). Since the CNV calling pipeline requires at least 5 consecutive probes before calling a region copy number variant, our theoretical resolution for CNV detection is 1465 bp (median spacing*4 + median oligo length*5).

### False positive rate

The false positive rate was calculated based on the 8 sex-mismatched arrays in this study: the length of chromosome × (from all the 8 hybs.) having a log2-ratio with a different signal than it should (given the sex-mismatched hybridization), dividing by the length of chrX multiplied by the number of sex-mismatched arrays (25,694,212/(88,516,663*8) = 3.62%). Since the FPR can be overestimated from sex-mismatched arrays, due to the assumption that no CNV exist in the chrX of sex-mismatched arrays, the FPR was also calculated from the self-self experiment and was determined as the length of sequence that would normally be called a CNV with our CNV calling pipeline. The FPR was determined as being 0.0085%.

### Enrichment analysis

Bovine segmental duplication (SD) data was retrieved from [89]. They used two independent approaches to detect segmental duplications: WGAC (whole-genome assembly comparison), which is a BLAST-based analysis of all assembled sequence that detects self alignments (>90% and 1 kb); and WSSD (whole-genome shotgun sequence detection), which is an assembly-independent approach that examines the reference sequence for an increase in WGS read depth-of-coverage. This strategy has been used previously to map SDs in the human [49] and mouse [27] genomes. From their global data we choose to filter out those SDs bigger than 94% identity using WGAC if they were not also confirmed by WSSD. The reason for this relates to the fact that the assembly of highly similar duplicated sequences will often be missed, collapsed or mis-assigned [90,91].

The association of CNVRs with genomic features (SDs, assembly gaps, genes and conserved elements) was tested by randomly permuting the genomic position of each CNVR 10 000 times and determining the sequence content of the resulting region or flanking regions.

### Real-Time PCR

Validation with RT-PCR was executed as previously described [47], with the Applied Biosystems 7900HT Sequence Detection System used for the Taqman assays, and downstream analysis performed with SDS 2.2 software. The full sequence of the CNVR was BLASTN-searched against the bovine genome sequence in order to identify a subregion that was unique and specific to the chromosomal location of the CNVR. PCR primers and probes were designed in this subregion of the CNVR using the ProbeFinder software from Roche Applied Science (Additional file 10, Table S8). Criteria for classifying a "not working" primer involved two parameters: reaction efficiency below 85%, and Pearson correlation of each standard curve below 0.95. Only ten of the original twenty bovine samples were used due to lack of DNA availability. For each target, the relative quantification analysis with a reference female sample was done to calculate estimated copy numbers of each sample.

### Data availability

The full data set and designs from the oligo array CGH experiments have been submitted to GEO [92] under the accession ID GSE18174.

## Authors' contributions

JF, BT, LEH and CB designed the project. JF performed data analysis and drafted the manuscript. BT planned the RT-PCR validation experiments. CB was the principal investigator of the project. All the authors have contributed to writing this manuscript and have read and approved the contents of the final submitted version.

## Supplementary Material

Additional file 1 **Excel file includes Table S1**. CNV regions detected and the number of CNVs per sample. Click here for file

Additional file 2 **Powerpoint file includes Figure S1**. Pedigree for the related animals. The numbers correspond to the sample ID, described in Table S1. Click here for file

Additional file 3 **Excel file includes Figure S2**. Correlation between chromosome length and number of CNVRs. Click here for file

Additional file 11 **Excel file includes Table S9**. Sequences in ChrUn that putatively belong to chromosome Y. Click here for file

Additional file 4 **Excel file includes Table S2**. CNVRs and ensembl genes within CNVRs. Click here for file

Additional file 5 **Excel file includes Table S3**. Cow-human ortholog genes with the respective dN and dS values (data from Ensembl database [52]). Click here for file

Additional file 6 **Text file includes Table S4**. Genes within CNV regions for which cow-human orthology exist and have a OMIM morbid ID [62]. Click here for file

Additional file 7 **Excel file includes Table S5**. Genes within CNV regions for which cow-human orthology exist and have been associated with a phenotype by a GWAS study. Click here for file

Additional file 8 **Excel file includes Table S6**. All CNVRs and QTLs within 2 Mb of a CNVR. Click here for file

Additional file 9 **Excel file includes Table S7**. All CNVRs and OMIA genes within 2 Mb of a CNVR. Click here for file

Additional file 10 **Excel file includes Table S8**. Primers and result plots for the RT-PCR experiments. Click here for file

Additional file 12 **Excel file includes Table S10**. Array CGH hybridizations. Extra information on the samples and experiment design is in GEO [92]. Click here for file
